# High-Precision Salt Concentration Detection Using a CMUT Array with Temperature Compensation

**DOI:** 10.3390/mi17040424

**Published:** 2026-03-30

**Authors:** Hanchi Chai, Changde He, Mengke Luo, Guojun Zhang, Hongliang Wang, Renxin Wang, Yuhua Yang, Jiangong Cui, Wendong Zhang, Licheng Jia

**Affiliations:** State Key Laboratory of Extreme Environment Optoelectronic Dynamic Measurement Technology and Instrument, North University of China, Taiyuan 030051, China

**Keywords:** temperature compensation, CMUT array, salt concentration detection, time-of-flight (TOF), ultrasonic velocity

## Abstract

This paper presents a miniaturized and highly accurate saltwater concentration monitoring system based on Capacitive Micromachined Ultrasonic Transducer (CMUT) array technology. The system incorporates a highly integrated CMUT array with a compact footprint of 5 mm × 5 mm, capable of both transmitting and receiving ultrasonic signals, which significantly contributes to the system’s miniaturization and portability. To ensure accurate compensation for temperature-dependent variations in sound velocity, a TA610A temperature sensor is integrated for continuous real-time monitoring of the salt solution temperature. By acquiring ultrasonic echo signals, the system calculates the time-of-flight (TOF) of the acoustic waves. Based on the TOF and real-time temperature data, the sound velocity is determined, and the salt concentration is subsequently derived with temperature compensation applied to enhance measurement accuracy. Experimental results show a measurement precision of 0.1% and a maximum absolute error of 0.02%, confirming the system’s high accuracy and robustness. Combining stability, reliability, and a compact real-time sensing design, the proposed CMUT-based system holds significant promise for practical deployment in various industrial and environmental monitoring scenarios.

## 1. Introduction

In the food processing and cold chain industries, saltwater-based freezing systems are widely used for the rapid cooling and preservation of perishable products. These systems primarily rely on sodium chloride (NaCl) solutions as coolants due to their low freezing points, cost-effectiveness, and superior thermophysical properties [1]. Maintaining NaCl solutions within an optimal mass fraction range is crucial to ensure efficient heat transfer, prevent coolant solidification, and minimize corrosion risks in heat exchange equipment [2,3]. Real-time monitoring of saltwater concentration has become increasingly vital in modern freezing systems, especially in tunnel freezers, immersion chillers, and large-scale cold storage facilities. Variations in NaCl concentration, caused by factors such as dilution, evaporation, or operational cycles, can significantly affect system performance and energy efficiency. Excessively high concentrations may lead to pipeline crystallization and scaling [4], while under-saturated solutions at low ambient temperatures may fail to provide sufficient antifreeze protection. As a result, reliable and continuous monitoring of saltwater concentration is essential for maintaining process stability, prolonging equipment lifespan, and ensuring compliance with food safety standards.

Currently, the conductivity method is widely used for detecting salt concentration [5], based on the principle that the conductivity of a salt solution is positively correlated with its concentration. This makes it the most common method for measuring salt concentration, but it has several drawbacks, including high sensitivity to temperature variations, which requires precise compensation, and a limited measurement range. In highly concentrated salt solutions, the conductivity increases at a decreasing rate, eventually reaching a saturation point, which leads to poor accuracy when measuring high concentrations of saltwater [6]. The electrodes in conductivity sensors are susceptible to contamination from impurities and scaling, which not only significantly affects measurement accuracy but also requires frequent maintenance, thereby increasing operational complexity and costs. Moreover, the invasive nature of this method further limits its practicality and convenience for continuous monitoring. As a result, there is a pressing need for alternative methods that can effectively address these issues.

Ultrasonic detection offers solutions to many of the challenges associated with traditional measurement methods. This technology has gained widespread application across various fields due to its non-invasive nature, high sensitivity, broad applicability, ease of operation, low cost, real-time performance, and safety, as well as its ability to provide detailed images and data [7]. With the advancement of Micro-Electro-Mechanical Systems (MEMS) technology, micro-machined ultrasonic transducers have become smaller, more cost-effective, and highly efficient. These transducers can be classified into two main types based on their operating principles: Capacitive Micromachined Ultrasonic Transducers (CMUTs) [8] and Piezoelectric Micromachined Ultrasonic Transducers (PMUTs) [9]. Although PMUTs offer the advantages of miniaturization, easy integration, and low power consumption [10,11,12], CMUTs exhibit a wider operating bandwidth and higher sensitivity in comparison [13,14,15,16,17,18]. Currently, CMUTs have been widely applied in fields such as medical imaging diagnosis and industrial non-destructive testing [19,20,21]. They also show great potential in emerging areas like wearable detection systems and AR/VR haptic feedback [22], and more notably, their unique value in the field of biochemical detection is gradually emerging—this set of advantages also makes them particularly suitable for real-time salinity detection in brine cooling pipelines [23]. Among these advantages, the high-sensitivity characteristic can accurately capture the subtle changes in ultrasonic propagation velocity caused by salinity fluctuations, which ensures detection accuracy. Second, the miniaturized design enabled by MEMS manufacturing technology allows the sensor to be deployed in situ inside pipelines, effectively overcoming the space constraints faced by traditional detection equipment. Additionally, the polyurethane matching layer bonded to the CMUT surface provides strong corrosion resistance to brine, thereby ensuring the long-term operational reliability of the sensor in such harsh corrosive environments.

**Figure 1:**
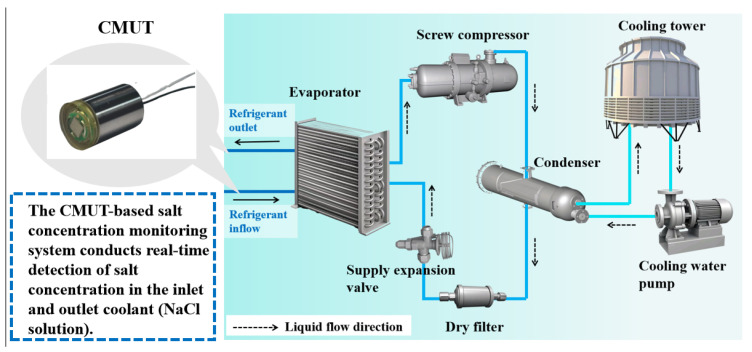
NaCl is used as a refrigerant in refrigeration systems.

In this work, a real-time salt concentration monitoring system was designed based on a 5 mm × 5 mm CMUT array. This system can be applied to a variety of real-time salt concentration testing environments. The host computer controls the CMUT array to transmit and receive acoustic wave signals. The acquisition board converts the collected mechanical signals into electrical signals. After temperature compensation, which is based on the temperature detected by the TA610A temperature sensor (Suzhou TASI, Suzhou, China), the flight time is calculated to obtain the salt concentration in the measured sample, which is then displayed on the host computer interface. As shown in Figure 1, NaCl is used as a refrigerant in refrigeration systems, demonstrating the relevance of accurate salt concentration measurement in such applications. This system offers a reliable salinity measurement solution that reduces cost while maintaining high accuracy, making it valuable for diverse salinity monitoring applications.

## 2. Design and Fabrication of CMUT Devices

### 2.1. Design

**Figure 2:**
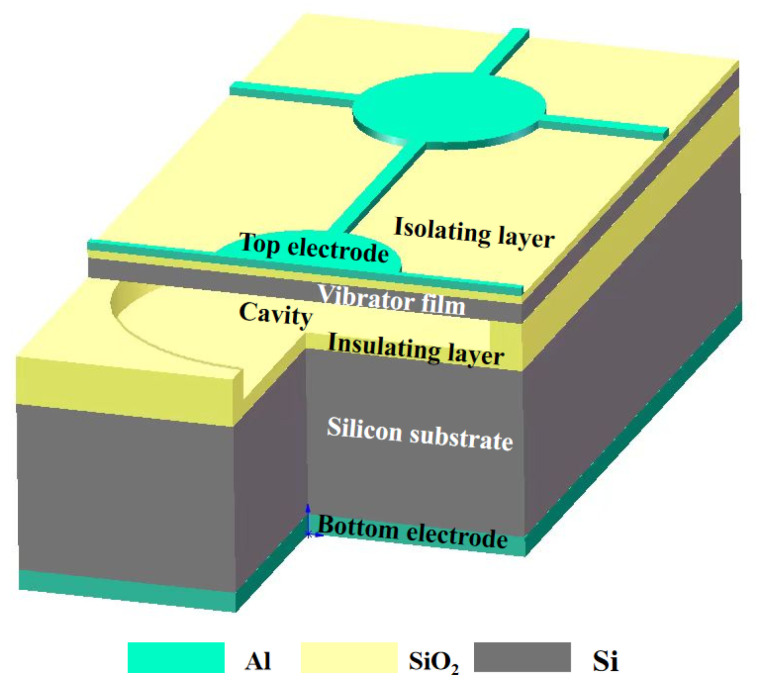
Basic structure of CMUT single sensing unit.

As shown in Figure 2, the structure of the CMUT consists, from top to bottom, of an upper electrode, isolation layer, vibrating membrane, insulating layer, silicon substrate, and lower electrode [24]. The CMUT array operates based on the principle of electrostatic capacitance. In the transmit mode, a bias voltage applied between the upper and lower electrodes generates an electrostatic force that causes the flexible membrane to deflect and vibrate, thereby producing ultrasonic waves through mechanical displacement. This process effectively converts electrical energy into mechanical energy, with the vibrational behavior of the membrane largely governed by its geometry and material properties. In the receive mode, incoming ultrasonic waves propagate through the medium and apply acoustic pressure on the membrane, inducing mechanical vibrations. These vibrations result in variations in the electrode gap, modulating the capacitance and generating an electrical charge. The resulting charge signal is then amplified and converted into a voltage signal by a charge amplifier circuit, enabling the conversion of mechanical energy back into electrical energy. Given the pivotal role of the diaphragm in determining CMUT array performance, optimization of structural parameters is crucial.

The resonant frequency of a CMUT in a vacuum is denoted as $f_{0}$. When the CMUT is immersed in a liquid with density $\rho_{L}$, its resonant frequency shifts to $f_{1}$:

(1) $$f_{0}=\frac{0.467h}{a^{2}\sqrt{\frac{E}{\rho_{M}\left(1-\sigma^{2}\right)}}}$$

(2) $$f_{1}=\frac{0.467h}{a^{2}\sqrt{\frac{E}{\rho_{M}\left(1-\sigma^{2}\right)}}}/\sqrt{1+0.67\rho_{L}a/\rho_{M}h}$$

where *h* is the membrane thickness, a is the membrane radius, *E* is the Young’s modulus of the membrane material, $\rho_{M}$ is the material density, and σ is the Poisson’s ratio of the membrane. It can be seen from the formula that the resonant frequency of the device is completely determined by its geometric dimensions and material properties. For a given membrane material, a larger radius or a smaller thickness results in a higher resonant frequency. To ensure the accuracy of concentration identification, the CMUT device designed in this study has a resonant frequency of 3 MHz. The structural parameters of the designed device are listed in Table 1.

### 2.2. Fabrication

**Figure 3:**
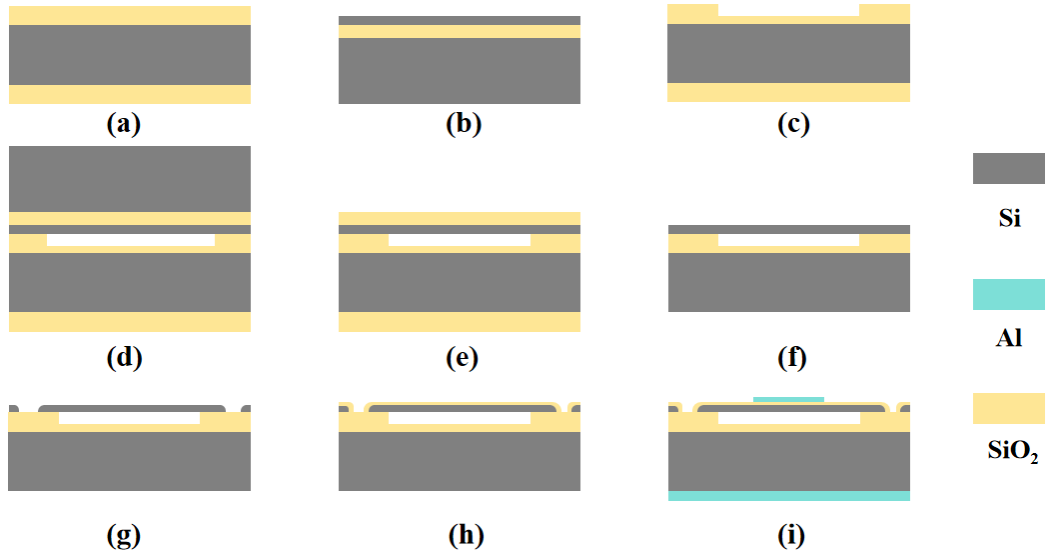
Fabrication process of the CMUT array using silicon wafer bonding technology. (**a**) Silicon oxide wafers. (**b**) SOI chip. (**c**) Cavity etching. (**d**) Bonding of silicon oxide wafers with SOI wafers. (**e**) Removal of silicon substrate. (**f**) Removal of the oxide layer. (**g**) Etching of isolation grooves and marking grooves. (**h**) Chemical vapor deposition of oxide layer. (**i**) Sputtering of the upper and lower electrodes.

The CMUT devices were fabricated using wafer bonding technology, which enables high-temperature direct bonding of two silicon surfaces, yielding strong adhesion and excellent hermetic sealing performance. The detailed fabrication process is illustrated in Figure 3.

First, 6-inch silicon oxide wafers and 6-inch SOI wafers were prepared, with a 400 nm thick silicon oxide layer on the silicon oxide wafers, as shown in Figure 3a,b. A circular cavity structure was formed by removing 300 nm of the oxide layer via reactive ion etching (RIE), while a 100 nm insulating layer was retained, as shown in Figure 3c. Subsequently, the SOI wafer and the patterned silicon oxide wafer were pre-bonded at room temperature under vacuum conditions, followed by annealing of the pre-bonded wafers at a maximum temperature of 1100 °C, as depicted in Figure 3d. After bonding, the buried oxide (BOX) layer and the handle substrate of the SOI wafer were removed by a combination of chemical mechanical polishing (CMP) and selective wet etching, exposing the device layer, as shown in Figure 3e,f. Next, isolation trenches and cavity structures were created on the front side of the wafer using deep reactive ion etching (DRIE), as illustrated in Figure 3g. Finally, after forming the SOI substrate with cavity structures, a 100 nm thick silicon dioxide insulating layer was deposited on the device layer. A patterned aluminum layer was sputtered to form the top electrode, while an unpatterned aluminum layer was deposited on the backside to serve as the bottom electrode, as shown in Figure 3h,i. A post-deposition thermal annealing was performed to establish ohmic contact between the bottom electrode and the highly doped silicon substrate, ensuring efficient electrical conduction.

**Figure 4:**
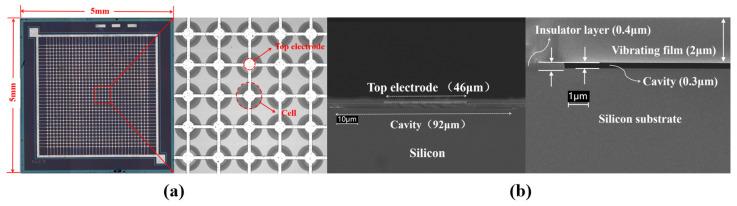
Optical microscope images of a fabricated CMUT. (**a**) Overview of the CMUT array and close-up view of a sensing cell in the CMUT array. (**b**) Cross-sectional electron microscopy (SEM SUPRA-55) (Shanghai ZEISS, Shanghai, China) image and partial enlarged view of the structure of a single sensing unit in CMUT.

The planar scanning electron microscopy (SEM) image of the fabricated CMUT is shown in Figure 4, featuring overall dimensions of 5 mm × 5 mm. Figure 4a shows the CMUT EM plane scan, and the EM cross-section scan of the CMUT individual array element is shown in Figure 4b.

**Figure 5:**
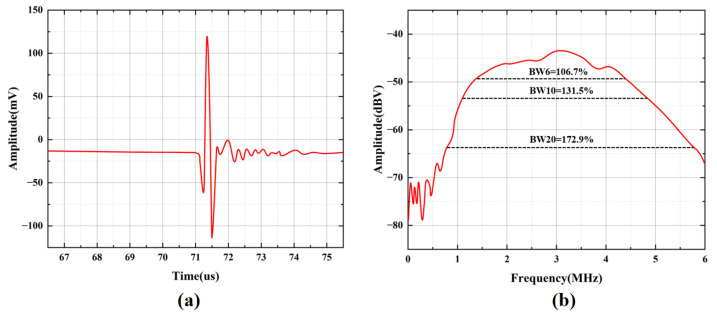
Bandwidth testing of CMUT device. (**a**) Echo signal diagram for bandwidth testing. (**b**) Bandwidth response plot of CMUT device.

The frequency response of the CMUT array was characterized in water at room temperature. The CMUT array and the aluminum reflection target were fixed on the same horizontal plane using a test fixture, facing each other. A broadband spike-pulse signal was generated by an ultrasonic pulser-receiver (OLYMPUS 5073PR) (Waltham OLYMPUS, Waltham, MA, USA) to drive the CMUT array for ultrasonic transmission and reception, and the received echo signals were amplified simultaneously. A DC bias voltage of 20 V was applied to the DC port of the CMUT array. An oscilloscope was used to record the measurement data. The echo signal waveform of the CMUT array is shown in Figure 5a, and the bandwidth results after Fourier transform are presented in Figure 5b.

The measured results show that the amplitude of the CMUT array is 218.42 mVpp, with a center frequency of 2.8925 MHz. The −6 dB bandwidth ranges from 1.35 MHz to 4.435 MHz, corresponding to a fractional bandwidth (FBW) of 106.7%. The −10 dB bandwidth ranges from 1.1 MHz to 4.905 MHz, with a fractional bandwidth of 131.5%. The −20 dB bandwidth ranges from 0.78 MHz to 5.78 MHz, with a fractional bandwidth of 172.9%. These results demonstrate that the CMUT array exhibits a wide bandwidth, which satisfies the design requirements.

**Figure 6:**
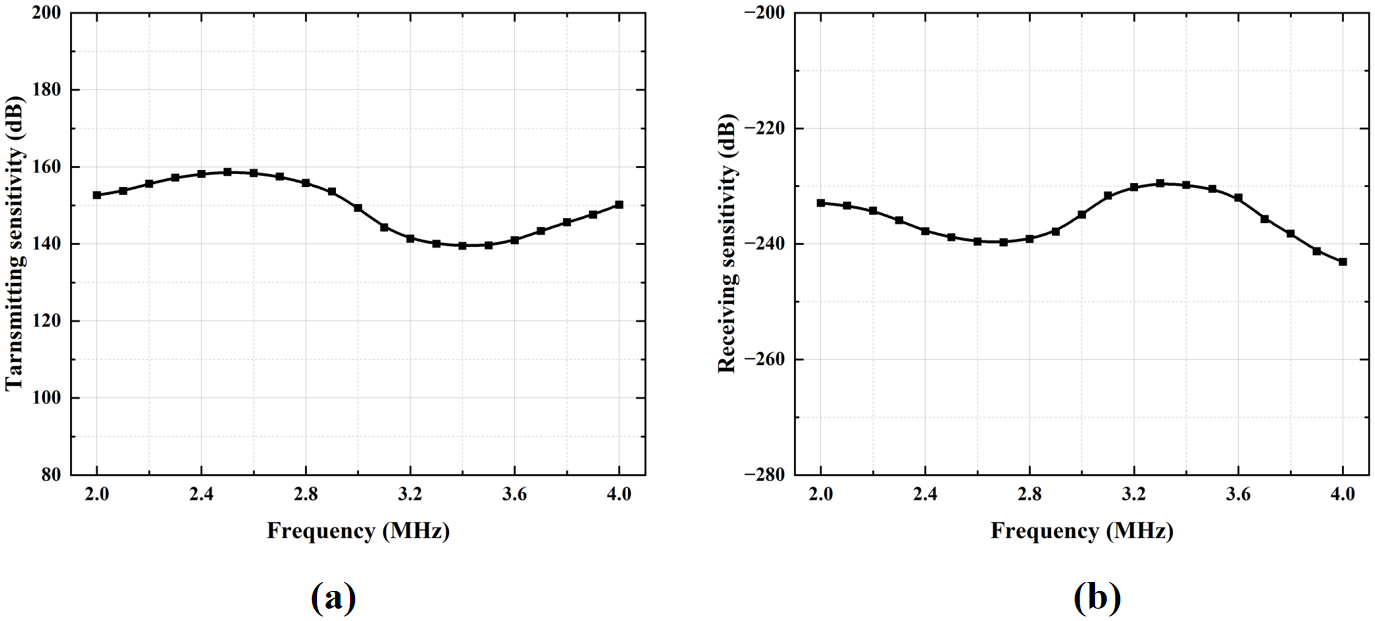
Sensitivity characterization of the CMUT device. (**a**) Transmit sensitivity curve. (**b**) Receive sensitivity curve.

Transmit and receive sensitivities are key parameters for evaluating the transmit and receive performance of CMUT linear array transducers. The transmitting sensitivity of the CMUT device was measured in a water tank at a distance of approximately 50 cm across a frequency range of 2 MHz to 4 MHz. As shown in Figure 6a, the transmit voltage response values of the standard transducer’s reflection response spectrum and the final transmit response spectrum at 3 MHz are 149.32 dB, which demonstrates that acoustic waves can be efficiently generated under the specified excitation conditions. To characterize the sensitivity, a reciprocal calibration method using a standard 3 MHz piezoelectric transducer as the reference was adopted. This method involves measuring the electrical output of the CMUT element in response to a known sound pressure over the same frequency range. As illustrated in Figure 6b, the receive sensitivity of both the standard transducer and the measured CMUT at 3 MHz is −234.93 dB, verifying the transducer’s ability to convert incident acoustic energy into high-fidelity electrical signals.

## 3. Methods

The detection of the concentration of saline solution by ultrasonic waves is achieved based on the characteristics of ultrasonic propagation velocity [25]. In a saline solution, the propagation velocity of ultrasonic waves is related to both the temperature and the salt concentration. Saline solutions with different concentrations exhibit unique characteristics of ultrasonic propagation velocity. This characteristic enables the ultrasonic detection technology to estimate the concentration value of the saline solution by measuring the propagation velocity of ultrasonic waves in the saline solution. By establishing the curve relationship between the standard concentration and the corresponding ultrasonic velocity, the rapid and accurate measurement of the concentration can be realized.

The TOF of ultrasonic waves is a key parameter in this test. By measuring the TOF, the propagation velocity c of the ultrasonic waves can be calculated in various media.

(3) c=d/t

In the formula, *d* represents the propagation distance of the ultrasonic wave, and *t* is the propagation time of the ultrasonic wave.

In this design, since the CMUT can independently transmit and receive signals, the acoustic wave undergoes two propagation paths over a distance *s* between the CMUT and the tested aluminum block.

(4) c=2s/t

Ultrasonic waves primarily propagate as compression waves in liquids, transferring energy through molecular interactions. During this process, liquid molecules experience periodic compression and rarefaction, resulting in the transmission of sound waves.

(5) $$c=\sqrt{\frac{K}{\rho }}$$

In the formula, *K* represents the elastic modulus of the medium, and ρ is the density of the medium. The adiabatic compressibility β is inversely proportional to the elastic modulus *K*; that is:

(6) $$c=\sqrt{\frac{1}{\beta \rho }}$$

The adiabatic compressibility reflects the ability of the medium to resist compression under adiabatic conditions, which embodies the rigidity of the substance [26]. The smaller the adiabatic compressibility is, the poorer the rigidity of the medium will be, and the more difficult it is to compress the medium, while the faster the sound velocity will be. In this experiment, the concentration of salt is expressed as the mass fraction of salt. Assuming that the salt is completely dissolved in water, the expression for the concentration of the salt solution can be written as follows.

(7) $$G=\frac{m_{s}}{m_{s}+m_{w}}$$

When the solution is pure water, the concentration of the salt solution is 0%, and at this time, the initial velocity in the medium can be expressed as Δc(T,G=0). In the solution, the sound velocity is simultaneously affected by both the concentration of the liquid and the temperature. A correlation function describing the sound velocity as a function of the solution’s temperature and concentration can be formulated as follows.

(8) c=f(T,G)

When conducting tests and research on the properties of a solution, it is often more accurate and convenient to measure the relative change in the physical quantity being tested. In this experiment, Δc(T,G) represents the relative change in sound velocity, and the velocity formula can be expressed as follows.

(9) c(T,G)=c(T,G=0)+Δc(T,G)

According to Formulas (6) and (9), Δc(T,G) can be expressed as follows:

(10) $$\Delta c\left(T,G\right)=\sqrt{\frac{1}{\beta \rho }-}c\left(T,G=0\right)$$

By keeping the temperature constant, Δc(T,G=0) is a constant. Taking the derivative of the relative change in velocity with respect to the concentration, one can obtain:

(11) $$\frac{d\Delta c(T,G)}{dG}=\frac{d\Delta c(T,G)}{d\beta }\cdot \frac{d\beta }{dG}+\frac{d\Delta c(T,G)}{d\rho }\cdot \frac{d\rho }{dG}$$

By substituting Formula (8) into the expression, the following result can be obtained.

(12) $$\frac{d\Delta c(T,G)}{dG}=-\sqrt{\beta^{3}\rho }\cdot \frac{d\beta }{dG}-\sqrt{\rho^{3}\beta }\cdot \frac{d\rho }{dG}$$

From the above formulas, it can be inferred that at a fixed temperature in Formula (7), the sound velocity only varies with the solution concentration. Therefore, a model of the relationship between the sound velocity and the concentration can be established. By using a thermocouple to conduct concentration gradient tests at different temperatures and performing temperature compensation based on reference tables [27], a model of the sound velocity in relation to both the temperature and the solution concentration can be established. Through the measurement of the temperature and the sound velocity, the concentration of the salt solution can be calculated and obtained in real time.

## 4. Detection System

The detection of the density of saltwater using ultrasonic waves is based on the ultrasonic TOF. When ultrasonic waves in water encounter a metal surface, due to the high density and sound speed of the metal, most of the acoustic energy is reflected back into the water. The acoustic impedance of the metal (the ratio of wave speed to density) is much higher than that of water, so the reflection coefficient is close to 1, meaning that almost all the acoustic energy is reflected [28]. In this experiment, the CMUT is placed facing an aluminum block. The CMUT is responsible for both the emission and reception of ultrasonic waves. Therefore, the flight distance of the ultrasonic waves is twice the distance from the CMUT to the aluminum block. The acoustic impedance of polyurethane is typically in the range of 1.5–3.0 MRayl, and that of saltwater is in the range of 1.5–2.0 MRayl. Thus, this material is well-matched with the aqueous solution in terms of acoustic impedance [29]. In this design, the device encapsulation material adopted is JA-2S cast polyurethane acoustically transparent rubber (Taiyuan SXCRI SynMat, Taiyuan, China). Experimental characterizations verify that this material has a sound velocity of 1500 m/s and an acoustic transmission coefficient higher than 90%, which is well matched to the salinity test environment.

**Figure 7:**
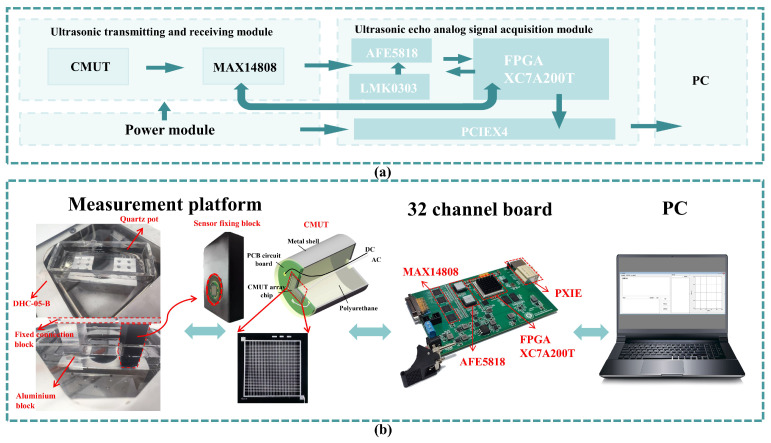
Schematic illustrations of the developed CMUT-based ultrasonic salt concentration detection system. (**a**) Functional block. (**b**) Measurement setup.

Figure 7a,b present the block diagram of each module and the test connection diagram of the proposed system, respectively. On the acquisition board, the FPGA (San Jose Xilinx, San Jose, CA, USA), MAX14808 (San Jose Maxim, San Jose, CA, USA), and AFE5818 (Dallas TI, Dallas, TX, USA) chips are responsible for control, signal transmission, and signal acquisition, respectively. The acquired data is processed and transmitted to the PC host via the PXIe interface for further analysis and storage. The MAX14808 generates pulsed square-wave signals to excite the CMUT for ultrasonic emission. After reflection, the CMUT receives the ultrasonic signal, and the TR switch in the MAX14808 is activated to route the signal to the AFE5818 chip. This chip integrates a low-noise amplifier (LNA), voltage-controlled attenuator (VCAT), programmable gain amplifier (PGA), low-pass filter (LPF), and a 14/12-bit analog-to-digital converter (ADC). The LNA provides variable gains of 12 dB, 18 dB, and 24 dB, while the PGA offers variable gains of 24 dB and 30 dB.

**Figure 8:**
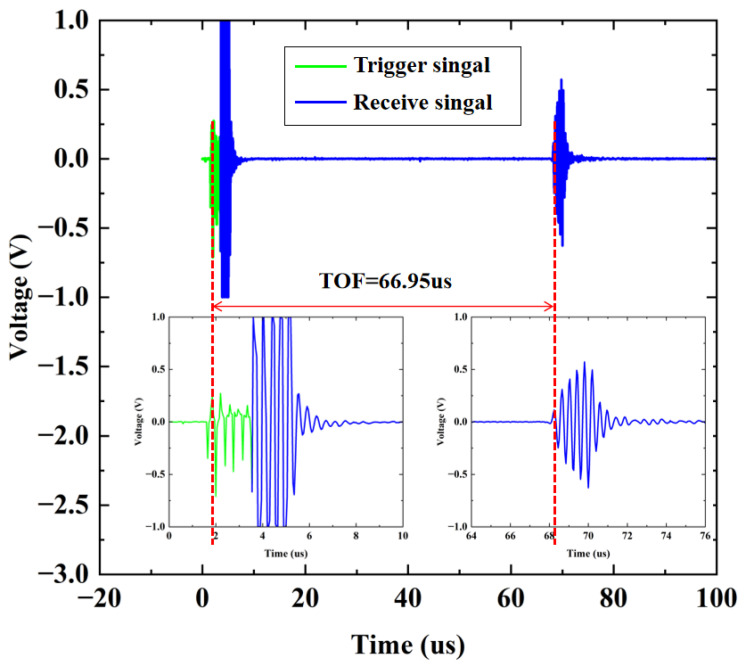
Calculation of the TOF.

During the testing process, the designed CMUT and the TA610A temperature sensor were fixed and immersed in a quartz container filled with the NaCl solution to be measured. The quartz container was then stably placed in a water-bath heating device to ensure that the temperature sensor could provide accurate temperature values for temperature compensation. An aluminum block was placed 5 cm in front of the single-element CMUT to reflect the echo signals. A dual-output multi-tracking power supply was connected to the board to supply ±10 V to the MAX14808 chip. In the working state, the MAX chip emitted five signal waves with a frequency of 3.0 MHz to excite the CMUT. In this work, the difference between the first peak of the transmitted pulse signal and the first peak of the received signal was recorded as the ultrasonic TOF. The total distance of the transmitted and reflected ultrasonic waves was 10 cm. As shown in Figure 8, in a pure-water environment at 20 °C, the TOF between the transmitted and received signals was recorded as 66.95 µs. According to Formula (3), the ultrasonic velocity at this time was calculated to be 1493.65 m/s, which was consistent with the theoretical value of 1496.7 m/s. The TOF was calculated by the host computer and displayed on it.

## 5. Results

To establish the correlation function among concentration, temperature, and ultrasonic velocity for versatile applications, the experimental design prioritizes fixing the solution concentration while systematically varying temperature for data acquisition. Specifically, the temperature range is set from 15 °C to 50 °C in 5 °C increments, and the concentration range spans 0% to 18% in 3% increments. This methodology ensures clear isolation of the individual effect of temperature on ultrasonic velocity, eliminating interference from concurrent concentration changes. As a result, the experimental outcomes accurately characterize the relationship between temperature variations and ultrasonic velocity, with real-time temperature readings recorded by the TA610 sensor. From a scientific and practical standpoint, fixing concentration while altering temperature is the optimal approach for this experiment. This method effectively separates variables, minimizes experimental errors, and aligns with real-world application requirements. Using NaCl as the experimental reagent, test solutions of target concentrations are prepared and placed in a quartz container, thermally stabilized by a water bath to maintain constant temperature. When the TA610 sensor indicates that the solution temperature matches the target value, the host computer is triggered to collect data and display waveforms in real time. By calculating the ultrasonic TOF, the corresponding ultrasonic velocity at each concentration-temperature pair is determined.

**Figure 9:**
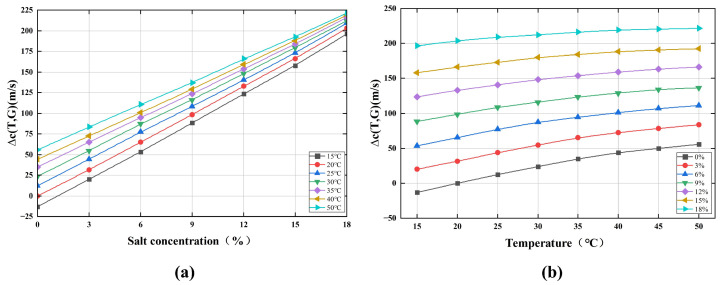
Diagram of experimental test results. (**a**) Measured ultrasonic velocity versus solution temperature of various salt solutions with different salt concentrations. (**b**) Measured ultrasonic velocity versus salt concentration of various salt solutions at different temperatures.

The measured data are presented as families of curves depicting ultrasonic velocity as a function of temperature and NaCl concentration, as shown in Figure 9a,b. The experimental plots reveal that ultrasonic velocity exhibits a nonlinear correlation with both NaCl concentration and temperature. In the low-concentration range of salt solutions, strong ion-water interactions enhance solution structural rigidity, leading to a rapid increase in bulk modulus with negligible density growth—changes that significantly elevate ultrasonic velocity. However, as salt concentration increases further, the saturation effect of hydrated ions attenuates the rise in bulk modulus, while density increases nearly linearly or accelerates slightly. This reduces the rate of change in K/ρ (bulk modulus over density), causing ultrasonic velocity to increase gradually. The result is a nonlinear, asymptotic relationship between velocity and concentration. Thus, a quadratic polynomial regression model was constructed to characterize this behavior.

Assuming the ultrasonic velocity variation is denoted as Δc(T,G), where *T* and *G* denote temperature and salt concentration, respectively, a predictive equation can be established as follows:

(13) $$\nu \left(T,C\right)=\beta_{0}+\beta_{1}T+\beta_{2}C+\beta_{3}T^{2}+\beta_{4}C^{2}+\beta_{5}TC+\varepsilon$$

In this equation, $\beta_{0}$ denotes the constant term (intercept); $\beta_{1}$ represents the linear coefficient for temperature; $\beta_{2}$ signifies the linear coefficient for salt concentration; $\beta_{3}$ is the quadratic coefficient for temperature; $\beta_{4}$ corresponds to the quadratic coefficient for salt concentration; $\beta_{5}$ serves as the coefficient for the temperature-concentration interaction term; and ε is the random error term.

The regression coefficients were estimated using the least squares method by constructing a feature matrix *X* and a target vector *C* from all experimental observations $\left(T_{i},G_{i},\Delta c_{i}\right)$, where each observation comprises temperature, salt concentration, and the corresponding ultrasonic velocity variation.

(14) C=Xβ+ε

The objective function of the least squares method is to minimize the sum of squared residuals, and the analytical solution is given by:

(15) $$\hat{\beta }={\left(X^{T}X\right)}^{-1}X^{T}C$$

**Figure 10:**
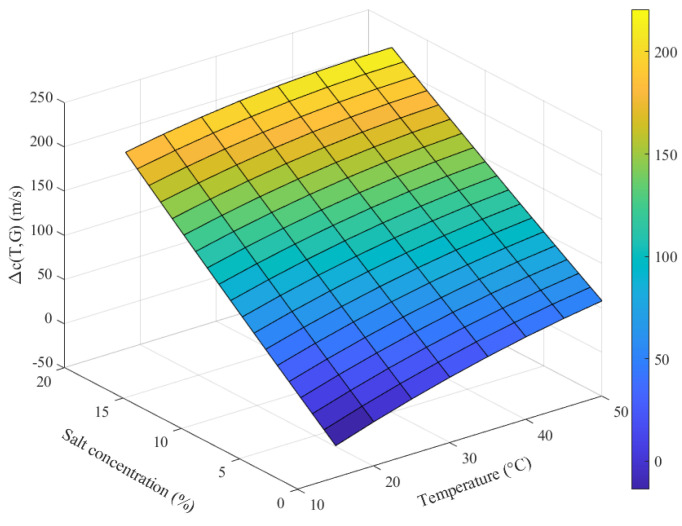
The fitted ultrasonic velocity surface.

**Figure 11:**
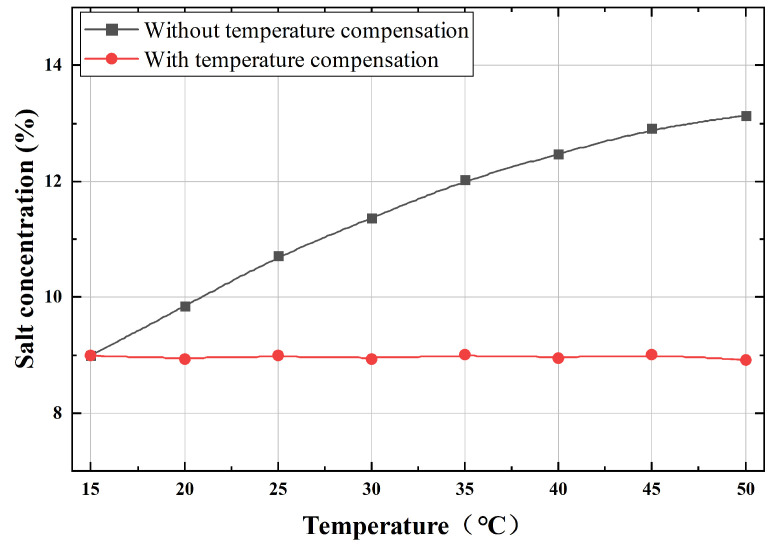
Comparison of salt concentration detection before and after temperature compensation.

Figure 10 shows the fitted ultrasonic velocity surface derived from the regression model. By inverting this surface to solve for NaCl concentration using measured velocity and temperature, the model enables temperature compensation to eliminate the effect of temperature on concentration detection. A validation test for temperature compensation was conducted using a 9% NaCl solution tested over a temperature range from 15 °C to 50 °C. The results of data processing with and without temperature compensation are shown in Figure 11. The results demonstrate that the designed NaCl concentration detection system stably and accurately compensates for temperature variations, effectively eliminating temperature effects on the measurement.

**Figure 12:**
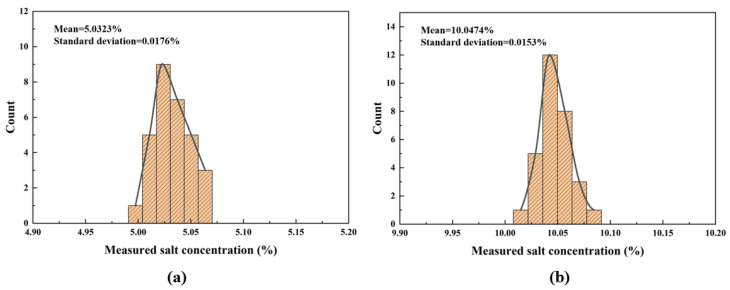
Multiple consecutive measurements of salt concentration. (**a**) Actual salt concentration of 5%. (**b**) Actual salt concentration of 10%.

To evaluate the accuracy of the proposed salt concentration monitoring system, 30 repeated measurements were performed for two solutions with target concentrations of 5% and 10%, respectively. The histograms of the measured salt concentrations at these two levels are shown in Figure 12. Calculations yield the following results: for the 5% NaCl solution, the mean concentration is 5.0323% (standard deviation = 0.0176%, relative error = 0.646% relative to the true value); for the 10% NaCl solution, the mean concentration is 10.0474% (standard deviation = 0.0153%, relative error = 0.474% relative to the true value). The data demonstrate that, across repeated trials, the measured standard deviation and relative error remain below 0.018% and 0.65%, respectively.

**Figure 13:**
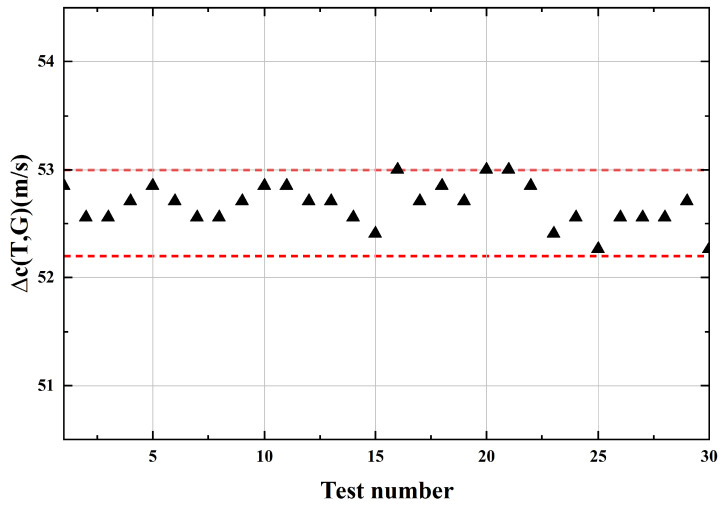
The ultrasonic velocity variation obtained from multiple measurements under identical test conditions.

To verify the stability of the designed NaCl concentration detection system, 30 ultrasonic signals were continuously acquired from a 5% NaCl solution at a constant temperature of 20 °C, as shown in Figure 13. The measured data fluctuated within a range of 0.7 m/s, corresponding to a salt concentration variation of 0.07%. These results indicate that the designed detection system exhibits excellent stability.

In this study, the salt concentration was derived from the ultrasonic velocity. Accordingly, the resolution of the developed salt concentration detection system can be defined as the minimum salt concentration that induces a detectable change in ultrasonic velocity. The ultrasonic velocity remains nearly constant when the sugar concentration varies by less than 0.1%. In contrast, a measurable change in ultrasonic velocity occurs when the salt concentration changes by 0.1% or more. Therefore, the proposed salt concentration detection system achieves a resolution as low as 0.1% for salt concentration measurement.

Table 2 compares this work with previously published studies and commercial salinometers. The designed NaCl concentration detection system outperforms traditional electrode-based salinometers in terms of measurement precision, error margins, and corrosion resistance. As a cost-effective alternative to laboratory-grade conductometric salinometers, it enables rapid, user-friendly, one-step measurements without requiring specialized expertise. In comparison to refractometric salinometers with limited testing ranges, this system offers a broader measurable concentration interval. Featuring a fast response time, the system supports real-time data acquisition: by integrating a high-precision temperature sensor and leveraging the embedded regression model, raw data are transmitted to a host computer. Here, NaCl concentration is automatically calculated and directly displayed on the user interface. This approach provides a robust solution for industrial NaCl concentration monitoring. The integration of the temperature acquisition module with the detection system is anticipated to yield a fully automated real-time monitoring system.

## 6. Conclusions

This work presents a salt concentration detection system based on a CMUT array. Experimental results demonstrate that the designed system exhibits a response time of less than 1 s. High-precision measurements are achieved within a concentration range of 0–18% and a temperature range of $15^{\circ }C$ to $50^{\circ }C$, with a measurement accuracy of 0.1% and a maximum error of 0.02%. The non-contact measurement principle not only ensures superior corrosion resistance but also effectively addresses the limitations of traditional contact-based sensors, which are prone to degradation and performance loss in saline environments. Thanks to the high integration and miniaturization offered by CMUT technology, the system exhibits substantial potential for real-time, portable, and corrosion-resistant concentration monitoring in industrial applications. Future work will focus on optimizing the temperature-concentration-sound velocity model combined with machine learning algorithms to further reduce measurement errors, as well as employing embedded systems to replace FPGAs for further cost reduction.

## Figures and Tables

**Table 1:** Detailed design parameters of the sensing cell in the CMUT.

Structural Parameters	Size
Device size (µm)	5000 × 5000
Number of cells	1024 (32 × 32)
Cell size (µm)	108 × 108
Vacuum chamber radius (µm)	46
Cavity height (µm)	0.3
Insulation height (µm)	0.1
Electrode radius (µm)	23
Film thickness (µm)	2
Electrode thickness (µm)	1

**Table 2:** Performance comparison of different salt concentration detection systems.

	Working Principle	Advantages/ Disadvantages	Characteristics
YSI ProDSS [30]	Conductivity	Advantages	1. High measurement accuracy (±0.1‰)
			2. Low tolerance (<0.2‰)
		Disadvantages	1. Narrow measuring range (0∼7%)
			2. Difficult to measure continuously (the electrode is prone to corrosion)
Horiba LAQUAtwin SALT-22 [31]	Ion-selective electrode	Advantages	Wide measurement range (0.1∼10%)
		Disadvantages	1. High tolerance (±0.1%)
			2. Difficult to measure continuously (the electrode is prone to corrosion)
vATAGO MASTER-S28 [32]	Light refraction	Advantages	High measurement accuracy (±0.2‰)
		Disadvantages	1. High tolerance (±0.2%)
			2. High requirements for operator
**This work**	Ultrasonic speed	Advantages	1. High measurement accuracy (0.1%)
			2. Low tolerance (<±0.2‰)
			3. Wide measurement range (0∼18%)
			4. Sustainable monitoring
		Disadvantages	Temperature compensation is required

## Data Availability

The data are available upon request from the authors.
